# Cross-Species Applications of Peptide Substrate Reporters to Quantitative Measurements of Kinase Activity

**DOI:** 10.1021/acsmeasuresciau.4c00030

**Published:** 2024-08-02

**Authors:** Mengqi Jonathan Fan, Misha Mehra, Kunwei Yang, Rahuljeet S. Chadha, Sababa Anber, Michelle L. Kovarik

**Affiliations:** Department of Chemistry, Trinity College, 300 Summit St., Hartford, Connecticut 06106, United States

**Keywords:** capillary electrophoresis, enzyme assay, peptide substrate reporter, kinase, phosphorylation

## Abstract

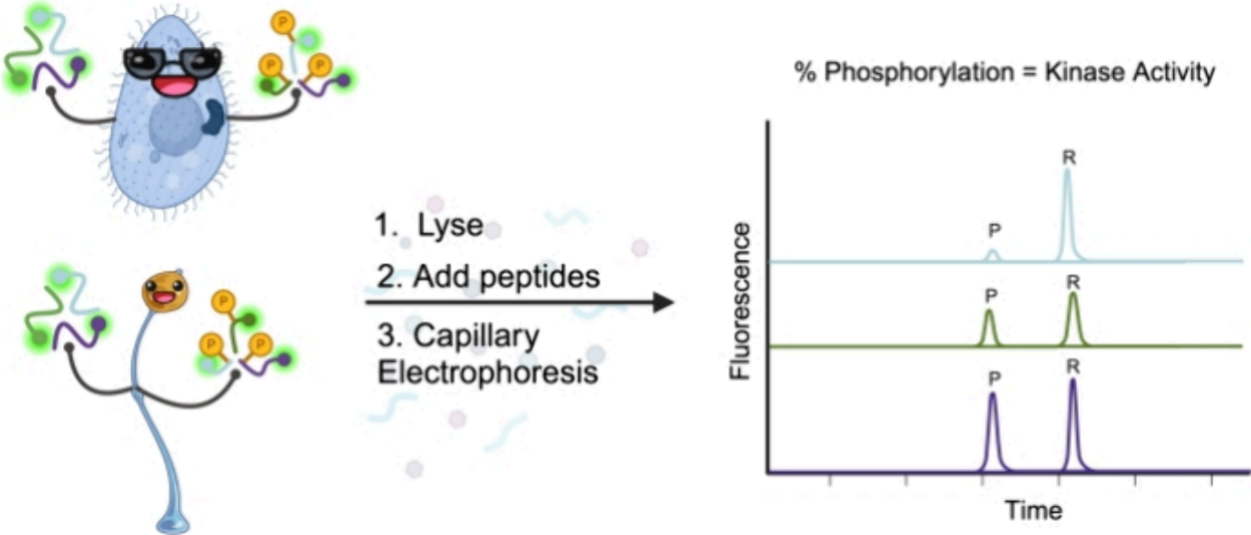

Peptide substrate reporters are short chains of amino acids designed to act as substrates for enzymes of interest. Combined with capillary electrophoresis and laser-induced fluorescence detection (CE-LIF), they are powerful molecular tools for quantitative measurements of enzyme activity even at the level of single cells. Although most peptide substrate reporters have been optimized for human or murine cells in health-related applications, their performance in nonmammalian organisms remains largely unexplored. In this study, we evaluated three peptide substrate reporters for protein kinase B (PKB) in two eukaryotic microbes, *Dictyostelium discoideum* and *Tetrahymena thermophila*, which are evolutionarily distant from mammals and from each other yet express PKB homologues. All three peptide substrate reporters were phosphorylated in lysates from both organisms but with varying phosphorylation kinetics and stability. To demonstrate reporter utility, we used one to screen for and identify the previously unknown stimulus needed to activate PHK5, the PKB homologue in *T. thermophila*. In *D. discoideum*, we employed the highly quantitative nature of these assays using CE-LIF to make precise measurements of PKB activity in response to transient stimulation, drug treatment, and genetic mutation. These results underscore the broad applicability of peptide substrate reporters across diverse species while highlighting the need for further research to determine effective peptide stabilization strategies across different biological contexts.

Peptide substrate reporters are molecular tools used to measure the activity of an enzyme of interest. Each reporter is a short (3–20 amino acid) peptide, often tagged with a fluorescent label, that is incubated in intact cells or lysates.^1,2^ For highly quantitative assays, electrophoretic separation is used to resolve the unmodified reporter from any enzymatic products, such as peptide fragments or phosphorylated peptide, and products are sensitively detected by laser-induced fluorescence (LIF). These experiments provide direct measures of enzyme activity and complement studies of enzymes based on mRNA transcript levels or antibody-based methods. Even when full-length, native substrates are known, peptide substrates have several advantages. Full-length protein substrates are typically quantified by Western blotting, but traditional implementations of this technique are labor-intensive, semiquantitative, and require relatively large quantities of samples and reagents, including validated antibodies against the protein of interest. Additionally, for enzymes such as kinases, total enzyme concentration is sometimes poorly correlated with enzyme activity, which is further regulated by post-translational modifications, localization, and other mechanisms. Peptide substrate reporters address these factors because modifications directly reflect net enzyme activity, and reporters and enzymatic products are readily separated by capillary electrophoresis (CE), a highly efficient, quantitative separation technique that is compatible with antibody-free analysis and small sample volumes, including single-cell analysis.^3^

Despite these advantages, peptide substrate reporters have not been as widely adopted for enzyme assays as more traditional methods. The development of peptide reporters is challenging because the amino acid sequence must be tailored to obtain stable substrates that have rapid reaction kinetics and are specific to an enzyme of interest. Peptide substrate reporters are often based on the sequence of a native substrate or the consensus sequence from several native substrates. However, because peptides are shorter than protein substrates, they are more susceptible to degradation by peptidases. Short peptides also lack secondary and tertiary structure, so they may have fewer favorable interactions with the enzyme of interest or undergo off-target interactions with related enzymes. For these reasons, peptide substrate reporter design is typically an iterative process involving both library screening and rational design or machine learning.^4−9^ Peptide substrate reporters have been developed for a range of enzymes, including kinases,^2,4,7,10−16^ phosphatases,^17^ deacylases,^18^ transferases,^9,19,20^ and many proteases. These substrates have typically been developed and validated using purified enzymes, lysates, or intact cells from humans or mice, and their applicability to other species is unclear. The advantages of peptide substrate reporters and the high investment in their development make it appealing to validate their application beyond mammalian cells for research in other organisms.

In this work, we evaluate three peptide substrate reporters for protein kinase B (PKB, also known as Akt) for measurements in two unicellular eukaryotes, the social amoeba *Dictyostelium discoideum* and the ciliate *Tetrahymena thermophila*. PKB is one of 39 primordial kinase families conserved throughout eukaryotic evolution.^21,22^ Thus, it is recognizably present across great evolutionary distances while maintaining high levels of sequence similarity (Figure 1). In human cells, the PI3K-PKB signaling pathway is associated with neutrophil chemotaxis, metabolism, and stress response and is commonly implicated in cancers.^23,24^ Because of its implications for human health, PKB has been a popular target for peptide substrate reporter research. All three peptide substrate reporters tested were initially developed and validated in human cell lines.^12,23,25−27^ Applications of these peptide substrate reporters have involved patient cells and mammalian cell lines for pancreatic cancer^28^ and rheumatoid arthritis.^29^ While previous work has focused on the role of PKB in human health, the PI3K-PKB pathway is integral to cell stress response in many organisms. In this work, we assess the suitability of three PKB reporters for enzyme assays in two organisms, *D. discoideum* and *T. thermophila*, demonstrating the applicability of peptide substrate reporters across evolutionary distance to biological investigations beyond human health.

**Figure 1:**
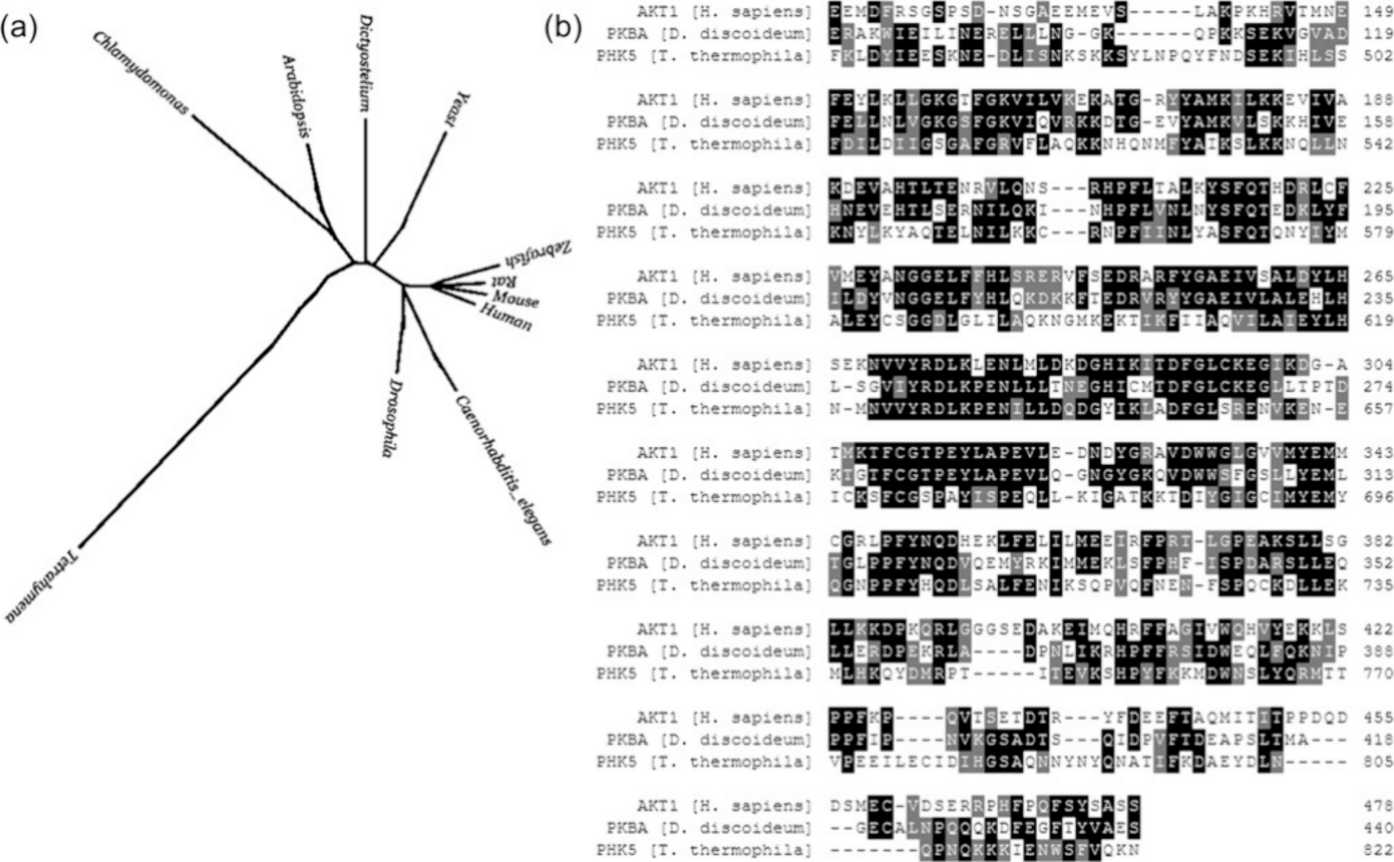
(a) Phylogenetic tree based on conserved proteins showing the evolutionary distance between human cells, the unicellular eukaryotes used in this study, and other common model organisms. Adapted with permission from ref (30). Copyright 2010 IEEE. (b) Sequence alignment of PKB homologues from human cells (AKT1, *H. sapiens*), *D. discoideum* (PKBA), and *T. thermophila* (PHK5).

## Methods

### Sequence Alignment

Protein sequences were obtained in the FASTA format from the NCBI database, aligned using T-Coffee,^31^ and formatted using Multiple Align Show from Bioinformatics.org.^32^

### Cell Culture

Axenic K-AX3 *D. discoideum* (DBS0236487) and mutant strain *cARA-* (DBS0236898) were obtained from the Dicty Stock Center.^33,34^ Cells were grown in shaking suspension at 180 rpm at 22 °C in HL-5 media supplemented with 13.5 g/L glucose, 300 μg/mL streptomycin and 100 μg/mL ampicillin and maintained at a cell density below 5 × 10^6^ cells/mL.^35^ HL-5 media was composed of 14 g/L proteose peptone no. 3 (BD), 7 g/L yeast extract, 3.5 mM dibasic sodium phosphate, and 11 mM monobasic potassium phosphate, pH 6.4–6.7. New cultures were started from frozen stocks every 2–3 weeks.

*T. thermophila* B2086.2 (SD00709) and CU428.2 (TSC_SD00178) strains were obtained from the *Tetrahymena* Stock Center,^36^ and cultured according to established protocols.^37^ Stock tubes were maintained in room temperature modified Neff media and replaced every 2–3 weeks. Modified Neff media was composed of 2.5 g/L proteose peptide, 2.5 g/L yeast extract, 5 g/L glucose, and 33.3 μM FeCl_3_. For experiments, cultures were expanded to 100,000–300,000 cells/mL in proteose peptone (PP) media in unshaken cultures at 30 °C.

### Peptide Substrate Reporters

Three peptide substrate reporters were obtained from Anaspec and stored at −80 °C as 500 μM aqueous stocks.

### Degradation Assays

Degradation assays were conducted as described previously.^38^ Briefly, aliquots of vegetative cells were washed twice, resuspended in ice-cold phosphate buffered saline at a density of 2 × 10^8^ cells/mL and lysed by three freeze–thaw cycles using liquid nitrogen. The resulting lysate was clarified by 5 min centrifugation at 15,000 × g, and the supernatant was stored at −80 °C. The total protein concentration was determined using a fluorescamine assay with a bovine serum albumin (BSA) calibration curve.^39^ Briefly, three parts diluted lysate (or BSA standard) in 30 mM borate buffer (pH 9) and one part 3 mg/mL fluorescamine in acetone were incubated for 5 min in the dark at room temperature. The fluorescence at 475 nm was then measured using 390 nm excitation. Based on this assay, the lysate was diluted to a concentration of 3 mg/mL in phosphate buffered saline. The degradation reaction was run at 25 °C and initiated by addition of 10 μM reporter to the diluted lysate. At fixed time points, aliquots were removed, and the reaction was stopped by incubation for 4 min at 95 °C.

### Phosphorylation in Lysates

For *D. discoideum* lysates, chemotaxis-competent cells were prepared as described previously.^40^ Briefly, cells were washed three times in ice-cold development buffer (DB; 10 mM phosphate buffer pH 6.5, 1 mM CaCl_2_, 2 mM MgCl_2_), resuspended at a density of 2 × 10^7^ cells/mL in room temperature DB, and shaken at 100 rpm. Prior to lysis, cells were basalated by addition of 4.3 mM caffeine for 20 min with 200 rpm shaking. Cells were then washed with ice-cold development buffer, stimulated with 1 μM cAMP, and lysed with an equal volume of 2× lysis buffer (50 mM MOPS, pH 7.6, 200 mM sodium chloride, 2 mM pervanadate, 50 mM glycerophosphate, 6 mM sodium pyrophosphate, 20 mM sodium fluoride, 2 mM EDTA, 2 mM EGTA, 4 μg/mL aprotinin, 4 μg/mL leupeptin, 2 mM DTT, 1% Triton X-100, and 20% glycerol).^41^ For assays with LY294002, cells were incubated with 15 μM LY294002 or an equivalent volume of DMSO as a vehicle control for 1 min immediately before cAMP stimulation and lysis.

For *T. thermophila* lysates, mating was induced by mixing equal cell numbers of B2086.2 and CU428.2 strains that had been starved for 16 h at 30 °C in unshaken Dryl’s buffer (2 mM phosphate buffer, 2 mM citrate, 1.5 mM CaCl_2_). Two hours after cells were mixed, microscopy was used to confirm that >75% of cells were paired. Samples were then lysed with an equal volume of ice-cold 2× lysis buffer, before and 30 s after addition of 20% proteose peptone stock to a final concentration of 2% or other stimuli as noted.

The protein concentrations of lysates were determined using the fluorescamine assay described above, and lysates were diluted to the same final concentration (0.4–0.6 mg/mL) in 1× lysis buffer. For *T. thermophila* stimulated with proteose peptone, it was not possible to determine protein concentrations in the cell lysate without interference from peptone, so these samples were diluted proportionately to unstimulated controls. Phosphorylation reactions were conducted in kinase buffer (25 mM MOPS, pH 7.4, 20 mM magnesium chloride, 25 mM glycerophosphate, 1 mM DTT).^41^ The peptide substrate reporter was added to a final concentration of 5 μM, and the reaction was started by addition of a final concentration of 2 mM ATP. The reaction was run at room temperature and stopped by the addition of CE run buffer. Negative control experiments were performed by substituting deionized water for ATP or denaturing the lysate with heat before the addition of peptide.

### Capillary Electrophoresis

Samples were analyzed using micellar electrokinetic chromatography (MEKC) in a PA-800 Plus capillary electrophoresis instrument (Beckman Coulter). For all assays of B-5/I, the CE run buffer was 100 mM borate, 100 mM SDS, pH 7.7, which was shown previously to resolve the reporter from all enzymatic products (i.e., phosphorylated peptide and fragments formed by cleavage of peptide bonds).^13^ The same buffer was used for phosphorylation assays of Crosstide. For degradation assays of Crosstide, the run buffer was 100 mM HEPES, 10 mM SDS, pH 8.0 since this buffer resolved all fragment peaks. For all VI–B assays, the run buffer was 100 mM borate, 15 mM SDS, pH 11.4. The capillary was 50 μm diameter bare silica, 21 cm effective length with an applied potential of 400 V/cm. Peaks for the unmodified reporters, the phosphorylated product, and some peptide fragments were identified by their migration times and confirmed by addition of purified standards. Peak integration was performed using 32 Karat (v. 10.1) software.

## Results and Discussion

In this work, we evaluated three peptide substrate reporters (Table 1) designed for human Akt for assays of PKB homologues in the eukaryotic microbes *D. discoideum* and *T. thermophila*. Crosstide is derived from the phosphorylation site of GSK3, a native PKB substrate,^26^ but is generically phosphorylated by other AGC kinases, including MAPKAP kinase-1, p70 S6 kinase, and PKA.^25,42^ B-5/I was developed using a screen to identify peptide sequences that would be specific for human PKB over MAPKAP kinase-1 and p70 S6 kinase.^25^ Then, the sequence for B-5/I was iteratively modified to make it resistant to peptidases by incorporating non-native amino acids while retaining a low *K*_M_ for human PKB.^13^*D. discoideum* and *T. thermophila* express the PKB homologues, PKBA and PHK5, respectively, during nutrient deprivation.^41,43^ Despite their sequence similarity to human PKB/AKT1 (Figure 1b), variations in sequence and structure were expected to change their phosphorylation activity toward the peptide substrate reporters. Promisingly, Crosstide has been used previously for phosphorylation assays of related AGC kinases, including an Akt homologue, in *S. cerevisiae*.^44−47^ However, the kinome of *S. cerevisiae* is closer to that of human cells than those of *D. discoideum* or *T. thermophila*.^21^

**Table 1:** Peptide Substrate Reporter Sequences^a^

**name**	**sequence**
Crosstide	5FAM-GRPRTSSFAEG
B-5/I	6FAM-GRPRAATFAEG-NH_2_
VI–B	6FAM-GRP**R**AFTF**A**-NH_2_

a Boldface underlined amino acids are *N*-methylated.

**Figure 2:**
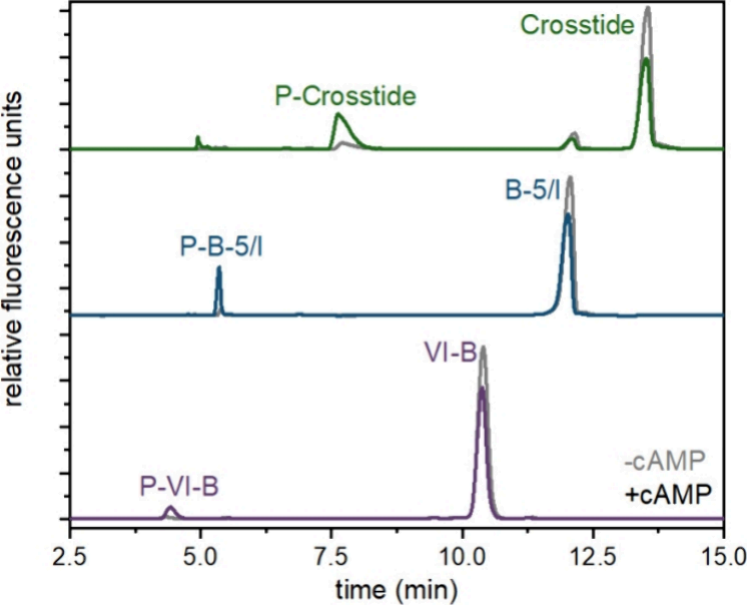
Phosphorylation of peptide substrate reporters in *D. discoideum* lysates. Electropherograms of the three peptide substrate reporters (Crosstide, green; B-5/I, blue; VI–B, purple) incubated with *D. discoideum* lysates from chemotaxis-competent cells unstimulated (gray) and stimulated (color) cells with cAMP. Cells were lysed just before or 20 s after stimulation. For all traces, *x*-axes have been normalized to align peaks for display only.

Thus, an initial step was to demonstrate phosphorylation of the reporters by enzymes from each organism. Purified, active enzymes from these organisms are not commercially available, and multiple attempts at custom protein expression of *D. discoideum* PKBA in various vectors were unsuccessful in yielding active enzyme. Consequently, we confirmed phosphorylation of the peptide substrate reporters in cell lysates from both unstimulated and stimulated cells that had been nutrient deprived (Figure 2). In *D. discoideum*, transient activation of PKBA coordinates chemotaxis and gene expression during social development, when individual cells form a differentiated, multicellular structure in response to nutrient deprivation.^48^ Much less is known about the kinome of *T. thermophila*. However, genome analysis identified 12 kinases containing a Pleckstrin homology; of these, PHK5 is most like mammalian Akt. Preliminary mRNA expression data show spikes in PHK5 levels 2 and 6 h after initiation of starvation-induced sexual reproduction (also called conjugation or mating).^43^

Lysates from *D. discoideum* demonstrated low levels of basal phosphorylation activity in unstimulated cells and increased phosphorylation activity toward all three reporters upon stimulation with cAMP (Figure 2). In *T. thermophila*, all three reporters were phosphorylated in lysates from unstimulated cells, but the phosphorylation activity was low and did not increase after initiation of mating as expected. PHK5 expression increases alongside the expression of the opposing phosphatase PTEN, suggesting that activation may be transient, as in *D. discoideum*.^43^ Thus, we hypothesized that an as-yet unidentified stimulus may be necessary to fully activate PHK5 and that activation may be transient due to concurrently high levels of PTEN.

**Figure 3:**
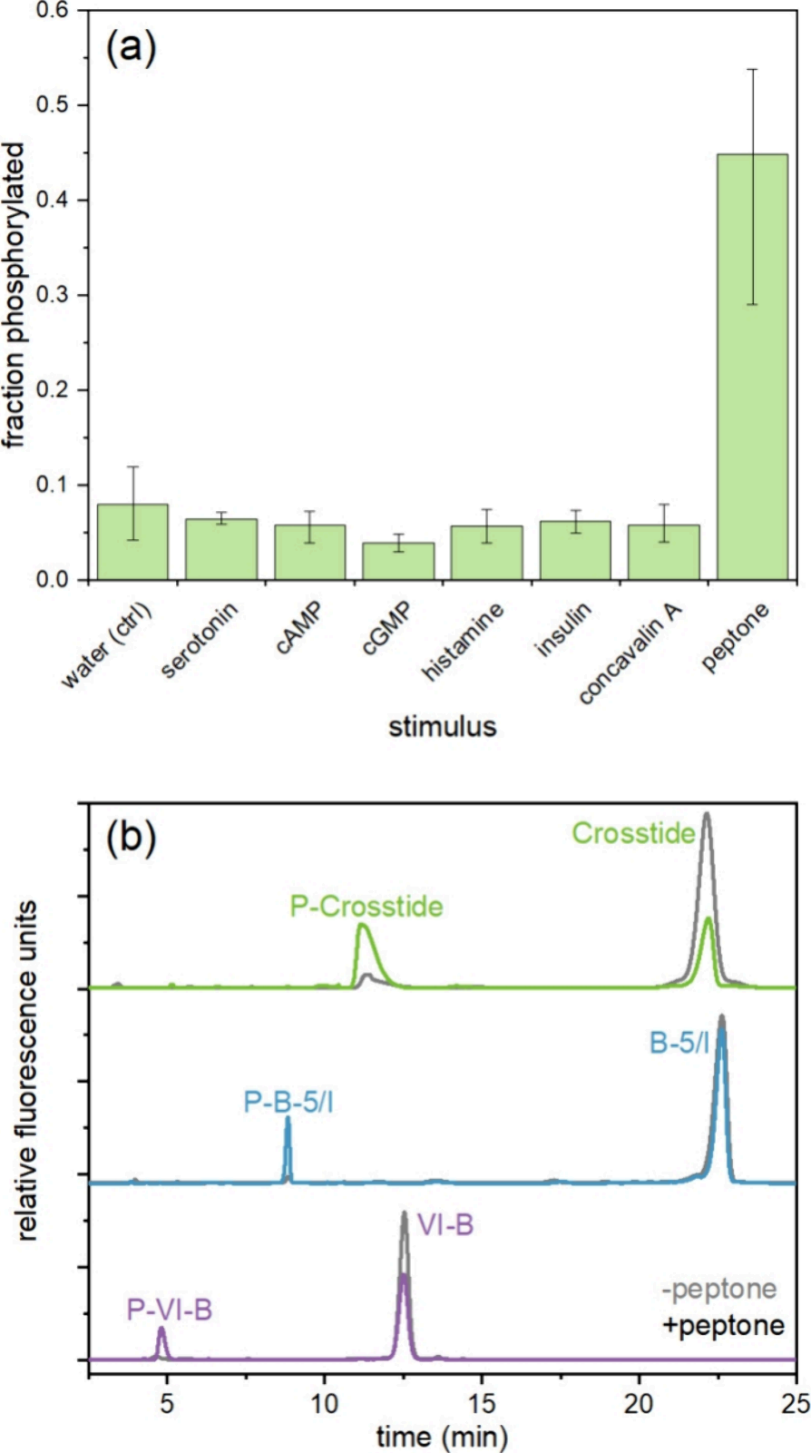
Phosphorylation of peptide substrate reporters in *T. thermophila* lysates. (a) Average fraction of Crosstide that was phosphorylated after 15 min incubation in lysates prepared 20 s after stimulation with 1 μM stimulus (or 2% for proteose peptone). Error bars represent the maximum and minimum values for *n* = 2–3 biological replicates on different days. (b) Electropherograms of the three peptide substrate reporters (Crosstide, green; B-5/I, blue; VI–B, purple) incubated with *T. thermophila* lysates from cells 2 h into conjugation for unstimulated (gray) and stimulated (color) cells with 2% proteose peptone. Cells were lysed just before or 30 s after stimulation. For all traces, *x*-axes have been normalized to align peaks for display only.

Using the peptide substrate reporter Crosstide, we screened several known hormones in *T. thermophila*, including cGMP, serotonin, histamine, insulin, and concavalin A.^49^ Cells were treated with a final concentration of 1 μM stimulus for 20 s prior to lysis. None of these stimuli resulted in increased phosphorylation activity (Figure 3a). (It is worth noting that mechanical agitation caused a slight increase in activity, and we modified our procedure to eliminate this interference.) Since conjugation is induced by nutrient deprivation, we then screened for the effect of nutrients on phosphorylation by adding proteose peptone to a final concentration of 2%. This treatment resulted in a dramatic increase in phosphorylation (Figure 3b). To our knowledge, this is the first assay of purported PHK5 activity, which has previously only been studied using mRNA expression,^43^ and the first indication that it may respond to nutrient levels. Because peptide substrate reporters are less specific than full-length native protein substrates, additional biochemical experiments are needed to confirm these results. However, these data demonstrate the utility of peptide substrate reporters for hypothesis testing and discovery. In particular, these experiments demonstrate the importance of analytical methods that do not rely on validated antibodies, which may not be available or which may not accurately reflect enzyme activity due to post-translational modifications and upstream signaling.

In addition to confirming that all three reporters were phosphorylated in lysates from both cell types, we also characterized the kinetics of the phosphorylation, which varied substantially between organisms. Using reactions with ≤10% total phosphorylation, we determined the phosphorylation rate for lysates from stimulated cells in units of zeptomoles of phosphorylated product per picogram of total protein per second (zmol pg^–1^ s^–1^; Table 2). Crosstide, which is based on the native substrate GSK-3,^26^ showed the fastest phosphorylation kinetics of the three peptides in both organisms. For *D. discoideum*, B-5/I was the next most preferred substrate. This kinetic trend is similar to that observed in human cells. Crosstide and a truncated version of B-5/I have similar values for *V*_max_ with human Akt1, but the *K*_M_ value is about 5-fold lower for Crosstide.^25^ The value of *V*_max_ has not been determined for VI–B with Akt1, but its phosphorylation rate is known to be slower than that of B-5/I.^13^ In contrast, *T. thermophila* lysates showed a preference for VI–B over B-5/I.

**Table 2:** Phosphorylation Rates of Each Reporter in Lysates from Each Cell Type 20–30 s after Stimulation with cAMP (*D. discoideum*) or Proteose Peptone (*T. thermophila*)

	phosphorylation rate (zmol pg^–1^ s^–1^)	
**peptide**	*D. discoideum*	*T. thermophila*
Crosstide	5.8 ± 0.8	9.1 ± 2.4
B-5/I	3.1 ± 0.6	1.8 ± 1.1
VI–B	1.4 ± 0.2	4.1 ± 0.9

Notably, in both microbes, initial phosphorylation rates for VI–B ranged from 1 to 4 zmol pg^–1^ s^–1^ after stimulation. These rates are much higher than rates obtained in intact human prostate cancer cells (LNCaP, 0.04 zmol pg^–1^ s^–1^)^6^ and pancreatic cancer cell lines (0.02–0.1 zmol pg^–1^ s^–1^)^28,50^ with the same reporter. Our assays were conducted in lysates containing phosphatase inhibitors, which will increase the apparent activity of PKB by removing the effects of any opposing phosphatases. Additionally, higher phosphorylation rates may arise from differing expression levels of PKB between cell types. For both *D. discoideum* and *T. thermophila*, PKB expression is linked to specific life cycle stages, and high levels of expression may be necessary to transduce short, transient signals.

### Application to Biochemical Assays in *D. discoideum*

**Figure 4:**
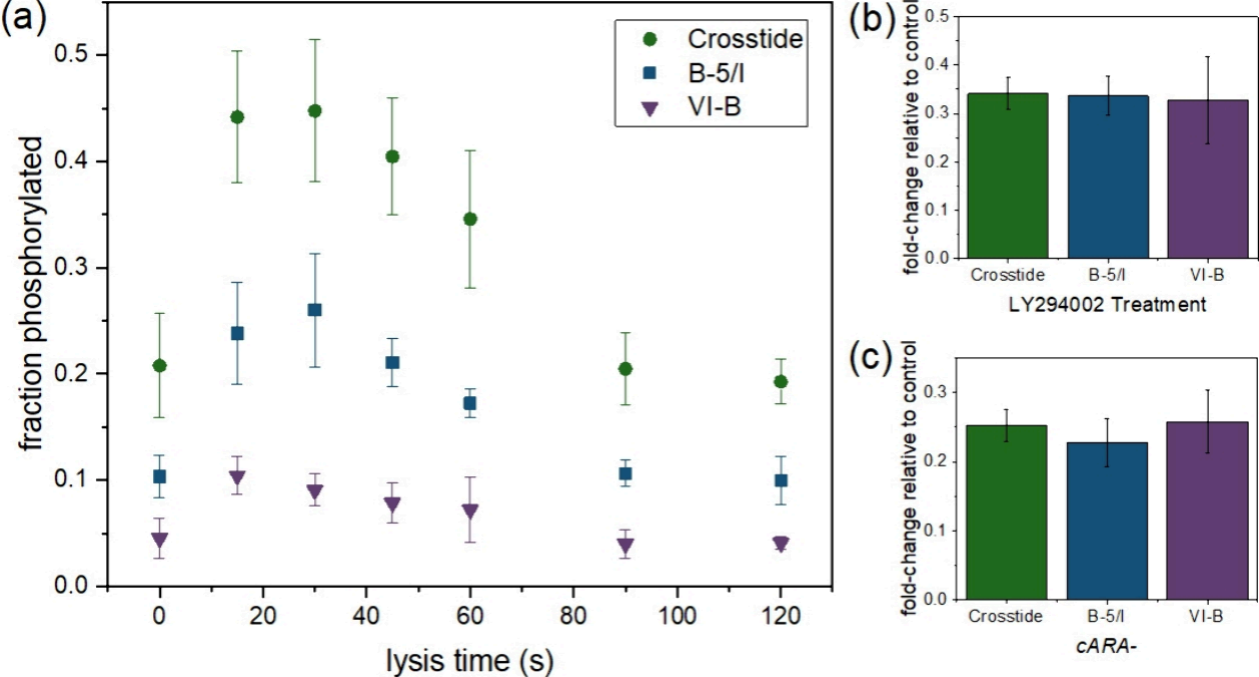
Confirmation of known changes to PKBA activity in *D. discoideum* using peptide substrate reporters. (a) Transient activation of kinase activity by cAMP stimulation. (b) Decreased kinase activity after treatment with LY294002. (c) Decreased kinase activity in *cARA*^*–*^ mutant cell lysates. For all experiments, social development proceeded for 1 h prior to stimulation and lysis. For (b) and (c) cells were lysed 15 s after stimulation with cAMP. Error bars represent the standard deviation of *n* = 3 biological replicates.

To further validate the use of these reporters in an evolutionarily distant organism, we conducted several quantitative biochemical assays of known physiological responses in *D. discoideum*. *D. discoideum* activates the PI3K-PKBA signaling pathway during chemotaxis in the early stages of its social life cycle.^51,52^ Under nutrient deprivation, *D. discoideum* undergoes a 24 h long social development process and transitions from free-living single cells into a differentiated, multicellular aggregate. Early in this process, cAMP secreted in pulses binds to a G-protein coupled receptor, cAR1, resulting in transient activation of the PI3K-PKBA pathway, which initiates actin polymerization and chemotaxis.^41^ We examined phosphorylation activity in relation to three aspects of this signaling pathway: the time scale of the transient activation after cAMP stimulus (Figure 4a); the pharmacological effect of the PI3K inhibitor LY294002 (Figure 4b); and genetic mutation to abolish the main isoform of the G-protein coupled receptor cAR1 (Figure 4c). In all three cases, results from peptide substrate reporters were in agreement with Western blotting results from our lab using an antibody against phospho-Akt substrates (Figures S1–S3) and from past research.^41^ However, the results from peptide substrate reporters highlight the highly quantitative nature of these assays using CE-LIF compared to semiquantitative Western blots.

For the first assay, we confirmed the rapid, transient increase in PKBA activity in response to cAMP stimulation. In *D. discoideum*, PKBA expression levels are high throughout the early stages of social development; however PKBA activity remains low until cAMP stimulation, which activates PI3K, resulting in a rapid, but transient, increase in PKB activity within 10–20 s that subsides 1–3 min poststimulus.^53,54^ Phosphorylation data for all three reporters reflected this transient activation. Reporters were maximally phosphorylated when cells were lysed 15–30 s after cAMP stimulation (Figure 4a), and the time course of activation was remarkably similar for all three peptides (Figure S4), even though the kinetics of phosphorylation differed, reflecting trends observed in Table 2.

Next, we tested pharmacological treatment of *D. discoideum*. We treated cells for 1 min with 15 μM LY294002, a PI3K inhibitor. LY294002 has been shown previously to reduce PKBA activity in *D. discoideum*.^53,55−57^ As expected, LY294002 treatment decreased phosphorylation activity toward all three reporters (Figure 4b). The magnitude of this effect was quite similar for all three peptides; percent phosphorylation of cAMP-stimulated cells decreased to 33–34% of the untreated phosphorylation levels upon LY294002 treatment. The LY294002 dose (15 μM) was previously identified as the EC50 for PKBA in *D. discoideum*,^53^ making this reduction in activity reasonable if PKBA is the enzyme primarily responsible for substrate phosphorylation.

Finally, we evaluated the ability of the peptide substrate reporters to reflect differences in signaling between “wild type” cells and a genetic mutant. The major G-protein coupled receptor for cAMP, cAR1, is deleted in the *cARA*^*–*^ strain, preventing cAMP stimulus from initiating PKBA activation. As expected, the *cARA*^*–*^ cell lysates showed minimal increase in phosphorylation activity toward the reporters in response to cAMP stimulation, resulting in phosphorylation levels roughly one-fourth of that observed for K-AX3 “wild type” controls (Figure 4c). As in past studies,^41^ phosphorylation activity was not completely abolished due to the presence of another cAR isoform, cAR3.

It is somewhat surprising that the results of these assays were so similar for the three peptides. If the peptides showed different levels of off-target phosphorylation by enzymes other than PKBA, then differences might be expected between peptides in their phosphorylation as a function of time, drug treatment, or mutation. Past research suggested that B-5/I was more specific for human Akt than Crosstide,^12,25^ but specificity may be one aspect of peptide substrate reporters that does not readily transfer between species. PKB is a member of the AGC group of small, cytoplasmic kinases, and the *Dictyostelium* kinome includes 21 kinases from this group, including the two PKB isoforms (PKBA and PKBR1) and an SGK ortholog.^58^ In *D. discoideum*, the PKBR1 isoform is highly homologous with PKBA,^55^ making it likely that any reporter peptide could be phosphorylated by both isoforms. Similarly, the *D. discoideum* SGK ortholog is likely to show activity toward the substrates since a previous screen of ∼50 kinases found that Crosstide and a truncated version of B-5/I were phosphorylated by SGK.^5^ However, the timing of PKBA expression during development differs from that of PKBR1 and SGK. Transcriptomic experiments show *pkbA* transcript levels peak during the first hour of development, then decline to basal levels by 4–6 h. In contrast, levels of *pkgB* transcripts (which code for PKBR1) and *DDB_G0277449* transcripts (which code for the SGK ortholog) only rise after 5 h of development and remain high at least through 12 h.^59^ Our experiments, conducted 1 h into social development, likely reflect PKBA activity over the activity of PKBR1 and SGK. Other AGC kinases may also contribute to phosphorylation of these reporters; however, evolutionary analysis of phospho-recognition sites shows that the R-x-x-T/S–F motif, associated with PKB substrates and present in the three peptides used here, is enriched in the human and yeast phosphoproteomes but not those of *D. discoideum* or *T. thermophila*.^60^ This may suggest that fewer kinases in these organisms recognize the motif. Nevertheless, further work with purified enzymes is required to evaluate specificity.

### Stability of the Reporter in Cell Lysates

**Figure 5:**
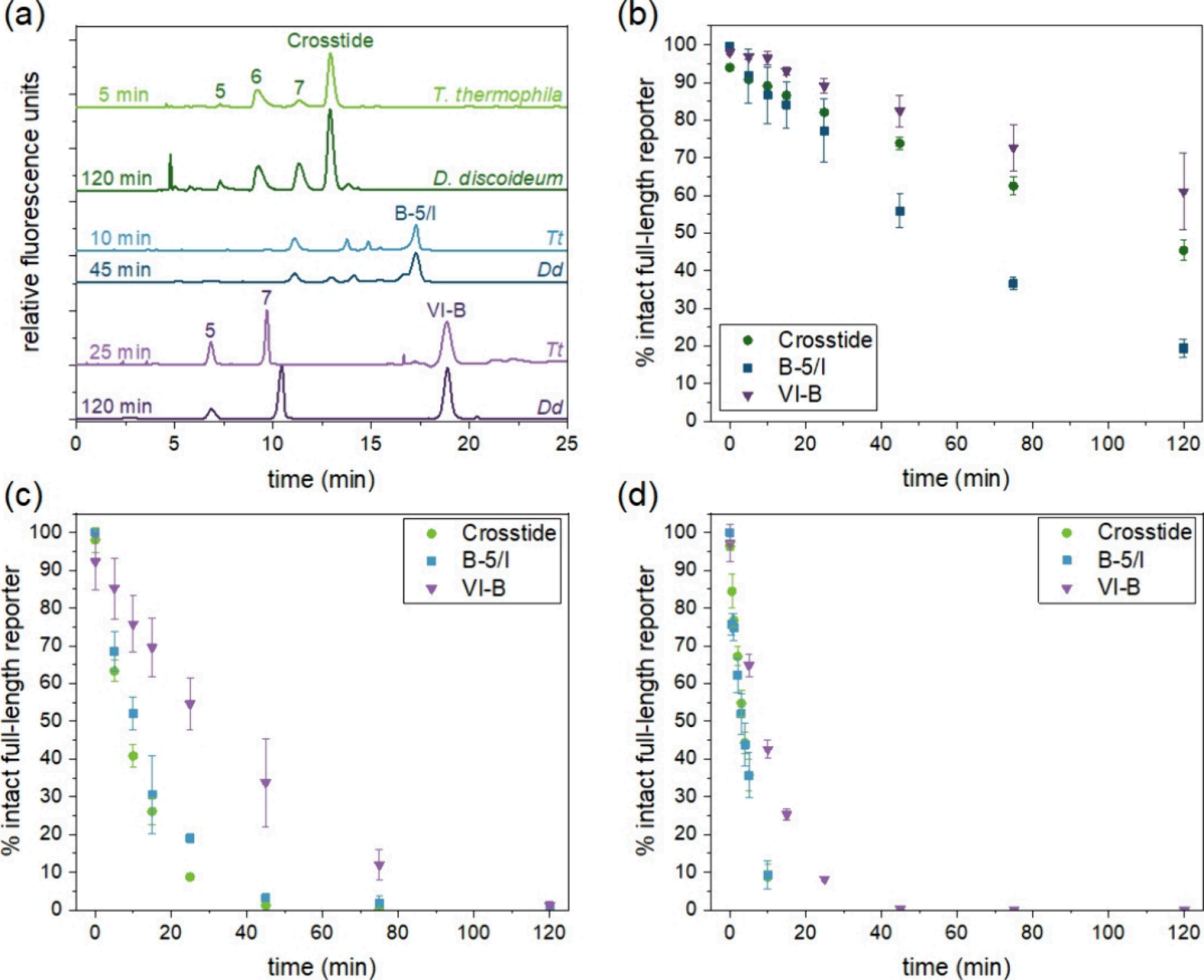
Peptide degradation data in freeze–thaw lysates. (a) Representative electropherograms of peptides incubated in vegetative cell lysates from *T. thermophila* B2086.2 (lighter) and *D. discoideum* (darker). Time points were selected to represent roughly one-half-life for each peptide (i.e., when the full-length reporter was approximately 50% degraded). Numbered peaks indicate peptide fragments identified by spiking with known standards and represented by the number of N-terminal amino acids remaining from the original unmodified reporter. (b-d) Degradation of the reporters over time in lysates from (b) *D. discoideum*, (c) *T. thermophila* B2086.2, and (d) *T. thermophila* CU428.2. Error bars represent the standard deviation for *n* = 3 biological replicates.

In lysates, peptide substrate reporters were used in combination with a cocktail of protease inhibitors to prevent degradation. However, application of peptide substrate reporters to advanced applications like single-cell analysis requires that they be resistant to degradation in intact cells. To check for stability, we incubated the reporters in freeze–thaw lysates from both organisms in the absence of protease inhibitors. We have previously shown that the stability of VI–B and its major degradation products varies widely across species.^38^ In the current work, we expanded our previous studies to include *T. thermophila* lysates and to look at degradation of B-5/I and Crosstide in addition to VI–B. We have previously shown that organisms that express different classes of proteolytic enzymes produce different fragmentation patterns.^38^ Genomic analysis of peptidase expression across organisms shows that *D. discoideum* and *T. thermophila* share the same core of eukaryotic peptidases despite their evolutionary distance.^61^ While fragmentation rates varied widely between *D. discoideum* and *T. thermophila*, the fragments observed were the same (Figure 5a). For Crosstide, the major fragments detected were those with 5, 6, or 7 amino acids remaining on the fluorescently labeled N-terminus, corresponding to 5FAM-GRPRT, 5FAM-GRPRTS, and 5FAM-GRPRTSS. For VI–B, the major fragments were 6FAM-GRP**R**A and 6FAM-GRP**R**AFT, where **R** is *N*-methylated arginine. Individual standards were not used to identify the fragments of B-5/I, but the peak patterns in electropherograms suggest that this peptide also formed the same fragments in lysates from both organisms. The observation of the same qualitative fragmentation patterns for all three peptides in both organisms supports the expectation based on genomic data that *D. discoideum* and *T. thermophila* express similar proteolytic enzymes.

To study degradation kinetics, we fit the data for each full-length peptide to a first-order reaction model and determined the half-life for each peptide for three biological replicates (Figure 5b-d, Table 3). Degradation rates and half-lives varied widely between organisms and peptides; however, some general trends were apparent. For all peptides, degradation was much faster in *T. thermophila* than in *D. discoideum*. *T. thermophila* is known to produce and secrete cysteine proteases and is a large (∼50 μm wide and ∼20 μm long), fast-growing cell. Its doubling time is nearly as rapid as that of *S. cerevisiae*, despite a cell volume that is ∼170-fold larger;^62,63^ however, our past research did not identify any correlation between VI–B degradation rate and the proliferation rate of the cells.^38^ Interestingly, the two strains used to produce mating pairs for phosphorylation experiments had different degradation kinetics; degradation was consistently 2–4 times faster in lysates from CU428.2 cells than from the B2086.2 strain. Further characterization of protease expression in these strains may be helpful identifying the enzyme(s) primarily responsible for degradation of these reporters.

**Table 3:** Half-Life of Each Reporter in Freeze-Thaw Lysates^a^

	half-life (min)		
	Crosstide	B-5/I	VI–B
*D. discoideum*	117 ± 10	51 ± 4	184 ± 65
*T. thermophila* B2086.2	8 ± 1	12 ± 4	22 ± 4
*T. thermophila* C428.2	2.9 ± 0.5	3.1 ± 0.5	6.7 ± 0.2

a Uncertainty values represent the standard deviation for 3 biological replicates.

Of the three peptides, VI–B had the longest half-life in both organisms. VI–B was optimized to be degradation-resistant in HeLa lysates through substitution of non-native *N*-methylated amino acids.^6^ This change appeared to improve its stability in the organisms studied here as well. That said, degradation of VI–B in *T. thermophila* was still very fast. In *D. discoideum* lysates, the initial rate of VI–B of fragmentation in lysates (based on the first 15 min of each reaction) was 0.3 ± 0.1 pmol of peptide per mg of total protein per second (pmol mg^–1^ s^–1^), In contrast, the initial degradation rate of VI–B in *T. thermophila* lysates (based on the first 5 min of each reaction) was 1.1 ± 0.5 pmol mg^–1^ s^–1^ for strain B2048.2 and 5.1 ± 0.3 pmol mg^–1^ s^–1^ for strain CU428.2. These rates are comparable to previous observations for peptide substrate reporter degradation (0.02–3 pmol mg^–1^ s^–1^),^28,29,38,64^ but the rate in *T. thermophila* is roughly an order of magnitude faster than in *D. discoideum*. Given the half-life of the peptides in *T. thermophila*, kinase assays in intact cells would likely require treatment with protease inhibitors to maintain intact reporter on the time scale of the experiment. Alternatively, a peptide substrate reporter could be designed for stability in eukaryotic microbes. As noted above, the 5-mer and 7-mer were prominent fragments of Crosstide and VI–B in both *D. discoideum* and *T. thermophila*. In human cells, non-native amino acids at position 5 yielded poor substrates for PKB, so this bond may be challenging to stabilize with compromising kinase activity toward the substrate. However, the bond between amino acids 7 and 8 has not previously been targeted for modifications because the 7-mer is not a major fragment in human cells.^13^ Our past research in *D. discoideum* has shown that formation of the 7-mer from the parent peptide has the highest rate constant of all possible fragmentation reactions and that formation of the 5-mer preferentially occurs via the 7-mer rather than from direct fragmentation of the parent peptide.^38^ Thus, stabilization of the bond between amino acids 7 and 8 represents a promising avenue for improved stability. Bulky N-terminal modifications and peptide cyclization or “stapling” are additional methods that may be employed.^65^

## Conclusions

Peptide substrate reporters are often developed and validated in a single cell type or a few cell types derived from the same species. However, these reporters could have broad applications across different organisms if their behavior in evolutionarily distant cell types were known or predictable. Here, we have demonstrated that three different peptide substrate reporters for the primordial kinase PKB are all well-suited to applications in evolutionarily distant organisms, suggesting that transfer of reporters to new species may be readily achievable. Peptide substrate reporters may be especially useful for cross-species comparisons because they are small and interact primarily with the enzyme’s active site. While antibodies may bind to epitopes that vary between organisms, the active site of most enzymes is highly conserved. As a result, reporter phosphorylation may be the most predictable and successful for peptide substrate reporters such as Crosstide that are based closely on native protein substrates.

In addition to enabling assays for enzymes that lack validated antibodies, peptide substrate reporters offer several additional advantages. CE-LIF detection is highly quantitative and facilitates precise measures of even small changes in enzyme activity relative to controls. Electrophoretic separation and LIF detection of peptide substrate reporters are also compatible with single-cell analysis.^8^ Even when antibodies are available, traditional Western blots require large numbers of cells and report on population-level changes in signaling, obscuring variation at the single-cell level. However, cell-to-cell variation in enzyme activity is critical in many biological processes.^3^ For example, during *D. discoideum* social development, genetically identical cells differentiate into stalk cells, which vacuolate and die, and spore cells, which enter a resting state until their dispersal to a more favorable environment.^66^ This profound difference in cell fate is based on differences in enzyme activity between cells, and validated tools to measure enzyme activity at the single-cell level are necessary to study the role of cellular heterogeneity in this process. Although the work presented here was conducted in cell lysates, peptide substrate reporters combined with capillary or microchip electrophoresis are a well-established method for single-cell enzyme assays.^3,65^ One challenge to single-cell assays with peptide substrate reporters is stabilizing reporters in intact cells, where degradation rates are an important consideration. As evidenced by the short half-life of VI–B in *T. thermophila* lysates, reporters that have been iteratively optimized in one species may not retain the same level of optimization in new species. Thus, further research is needed to determine which peptide stabilization strategies are effective across species. A deeper understanding of how peptide substrates behave in evolutionarily diverse cells will facilitate the full breadth of applications of these tools from basic biology to human health.

## Supporting Information Available

Procedure and results for Western blot experiments (Figures S1–S3) and normalized data for Figure 4a (Figure S4) (PDF)
