# Supplementary material for: Cross-Species Applications of Peptide Substrate Reporters to Quantitative Measurements of Kinase Activity
Source: ACS Meas Sci Au. 2024 Aug 2;4(5):546–55. doi: 10.1021/acsmeasuresciau.4c00030 (PMC11487760; doi:10.1021/acsmeasuresciau.4c00030)
Supplement: Supplementary file 1 — tg4c00030_si_001.pdf [file tg4c00030_si_001.pdf]

Supporting Information

Cross-Species Applications of Peptide Substrate Reporters to Quantitative Measurements of Kinase Activity

Mengqi Jonathan Fan,[a†#] Misha Mehra,[a†] Kunwei Yang,[a§] Rahuljeet S. Chadha,[a$] Sababa Anber,[a] and Michelle L. Kovarik[a]*

[a]Department of Chemistry, Trinity College, 300 Summit St., Hartford, CT 06106 USA

*Email: michelle.kovarik@trincoll.edu

†MJF and MM contributed equally to this paper.

Procedure for Western blotting 2

Figure S1. Effect of lysis time (Western blot)..3

Figure S2. Effect of LY294002 treatment (Western blot) 4

Figure S3. Effect of *cARA*- mutation (Western blot) 5

Figure S4. Normalized data for Figure 4a..6

Procedure for Western blotting. Western blotting for phosphorylated PKB substrates from *D. discoideum* lysates was performed as described previously.[1] Cells were lysed in 5× SDS lysis loading buffer (250 mM Tris·HCl, pH 6.8, 10% SDS, 30% v/v glycerol, 10 mM DTT, 0.05% w/v bromophenol blue). Lysates and a standard protein ladder (Thermo Scientific™ PageRuler™ Plus, 10-250 kDa) were loaded onto a precast 4 to 12%, Tris-Glycine gel (Invitrogen™ Novex™ WedgeWell™) and run at 220 V in running buffer for 35-40 min. Bands were transferred to PVDF membrane, incubated with blocking buffer (5% w/v nonfat dry milk in TBST) for 1 h, with primary antibody (Phospho-Akt Substrate (RXXS*/T*) (110B7E) Rabbit mAb, Cell Signaling Technology) overnight, and with secondary antibody (HRP-linked, anti-rabbit IgG, Cell Signaling Technology) at room temperature for 1 h. ECL reagent (Thermo Scientific/Pierce) was then added, and chemiluminescence was recorded using an Azure Biosystems Imaging System. Intensities of the protein bands were quantified using ImageJ.

[1] Y. Kamimura, M. Tang, and P. Devreotes, *Methods Mol. Biol. Clifton NJ*, 2009, *571*, 255.

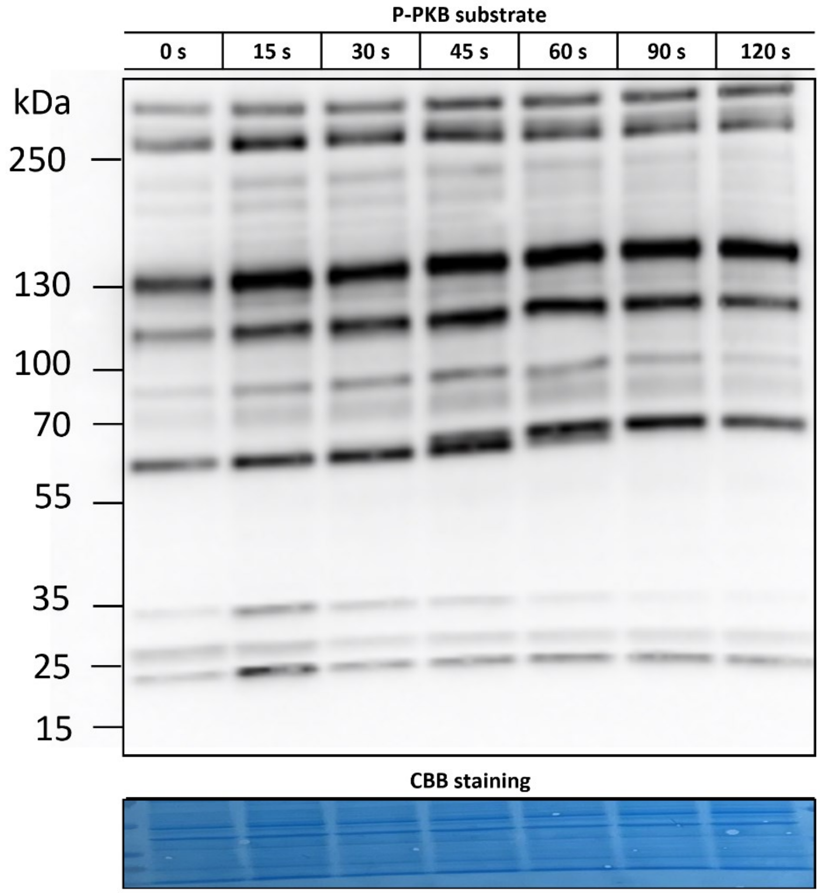

Figure S1. Western blot for samples in which cells were lysed at time points 0-120 s after cAMP stimulus.

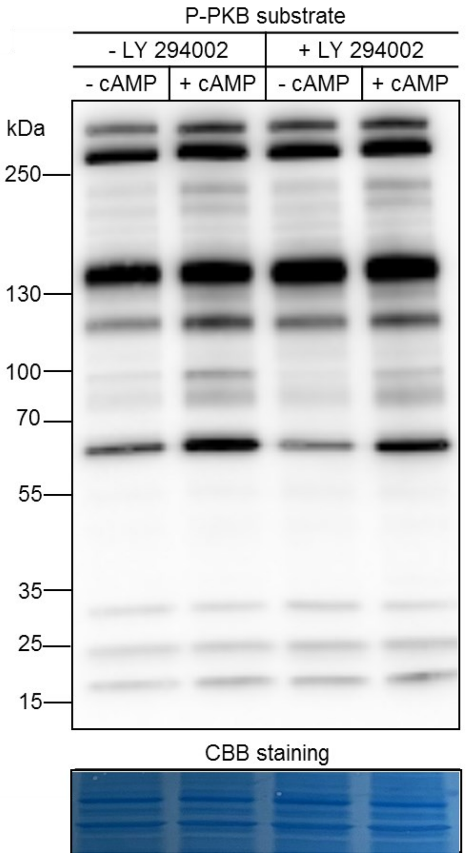

Figure S2. Western blot for cell lysates treated with LY294002 and stimulated with cAMP 15-20 s prior to cell lysis along with negative and positive controls.

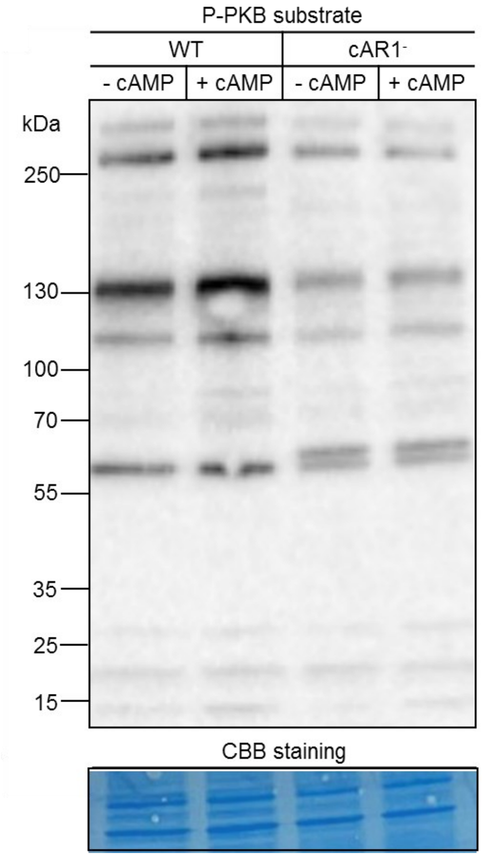

Figure S3. Western blot for cell lysates from wild-type (WT) K-AX3 cells and *cARA*- mutant cells that lack the G-protein coupled receptor cAR1.

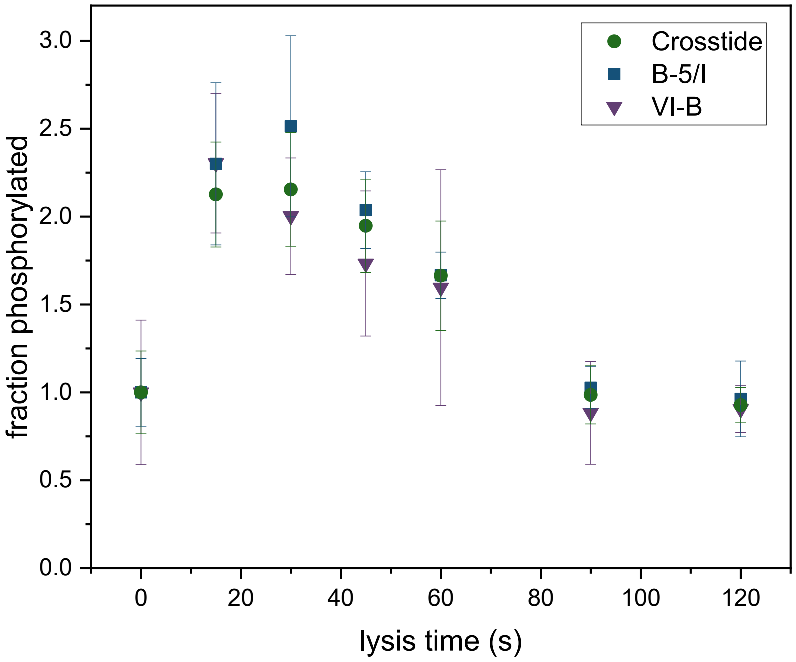

Figure S4. Phosphorylation data from Figure 4a presented with each peptide's phosphorylation level normalized to the level observed at time = 0 s, prior to stimulus with cAMP.
